# Violence, discrimination, and sexual health practices among adolescent men who have sex with men, transgender women and *travestis* in three cities in Brazil

**DOI:** 10.1590/0102-311XEN142922

**Published:** 2023-12-08

**Authors:** Marcelo Ryngelblum, Alexandre Grangeiro, Eliana Miura Zucchi, Marcia Thereza Couto, Ines Dourado, Laio Magno, Unaí Tupinambás, Maria Fernanda Tourinho Peres

**Affiliations:** 1 Faculdade de Medicina, Universidade de São Paulo, São Paulo, Brasil.; 2 Programa de Pós-graduação em Saúde Coletiva, Universidade Católica de Santos, Santos, Brasil.; 3 Instituto de Saúde Coletiva, Universidade Federal da Bahia, Salvador, Brasil.; 4 Departamento de Ciências da Vida, Universidade do Estado da Bahia, Salvador, Brasil.; 5 Faculdade de Medicina, Universidade Federal de Minas Gerais, Belo Horizonte, Brasil.

**Keywords:** Violence, Sexism, HIV, Adolescent, Self Care, Violência, Sexismo, HIV, Adolescente, Autocuidado, Violence, Sexismo, VIH, Autocuidado

## Abstract

The HIV epidemic has a disproportionate impact on adolescent and young men who have sex with men (AMSM) and transgender women and *travestis* (ATGW), with an increased HIV prevalence over the last 10 years. Violence affects the lives of these populations, undermining their ability to self-care and making them more vulnerable to HIV infection. In this study, we aimed to examine the association between different types of victimization by violence and discrimination and sexual health practices of these adolescent populations in steady and casual relationships. We conducted a cross-sectional study using baseline data from the cohort of PrEP1519 project. We used the mean score of sexual health practices as our outcome and the cumulative score of discrimination (within family, community, education, religious, online and public spaces) and violence (physical, sexual and intimate partner) as our exposure variable. We performed linear regression analyses to estimate the association between exposure and outcome. We found that 90% of AMSM and 95% of ATGW experienced at least one form of violence in the three months prior to this study and about 45% of ATGW suffered sexual violence during the same period. Experiencing discrimination within healthcare settings (from facilities or providers) was negatively associated with sexual health practices. Discrimination and violence negatively affect sexual health practices. HIV prevention and care of AMSM and ATGW people should involve listening to their experiences and addressing discrimination and violence in this population.

## Abstract (pt)

A epidemia de HIV afeta desproporcionalmente homens adolescentes que fazem sexo com homens (AHSH) e mulheres transgênero e travestis. Nos últimos 10 anos, a prevalência do HIV aumento. A violência afeta a vida dessas populações adolescentes, comprometendo sua capacidade de autocuidado e tornando-as mais vulneráveis à infecção pelo HIV. Este estudo buscou examinar a associação entre diferentes tipos de vitimização por violência e discriminação e práticas de saúde sexual entre essas populações adolescentes em relacionamentos estáveis e casuais. Realizamos um estudo transversal usando dados basais do estudo de coorte PrEP1519. Usamos o escore médio de práticas de saúde sexual como desfecho e o escore cumulativo de discriminação (familiar, comunitária, educacional, religiosa, *online* e em espaços públicos) e violência (parceiro físico, sexual e íntimo) como variável de exposição. Realizamos análises de regressão linear para estimar a associação entre exposição e desfecho. No todo, 90% dos AHSH e 95% dos mulheres transgênero e travestis sofreram pelo menos uma forma de violência nos três meses anterior à pesquisa e cerca de 45% dos mulheres transgênero e travestis sofreram violência sexual no mesmo período. Vivenciar discriminação dentro de ambientes de cuidados de saúde (instituições ou provedores) foi negativamente associado às práticas de saúde sexual. A discriminação e a violência afetam negativamente práticas de saúde sexual. A prevenção contra o HIV e o cuidado a AHSH e mulheres transgênero e travestis devem escutar suas experiências e enfrentar a discriminação e da violência nessa população.

## Abstract (es)

La epidemia del VIH afecta desproporcionadamente a los hombres adolescentes que tienen sexo con hombres (AHSH) y las mujeres transgénero y travestis. En los últimos 10 años, la prevalencia del VIH aumento. La violencia afecta la vida de estas poblaciones adolescentes, comprometiendo su capacidad de autocuidado y haciéndolas más vulnerables a la infección por VIH. Este estudio buscó examinar la asociación entre los diferentes tipos de victimización por violencia y discriminación y las prácticas de salud sexual entre estas poblaciones adolescentes en relaciones estables y casuales. Realizamos un estudio transversal utilizando datos basales del estudio de cohorte PrEP1519. Utilizamos el puntaje promedio de las prácticas de salud sexual como resultado y el puntaje acumulativo de discriminación (familiar, comunitario, educativo, religioso, en línea y en espacios públicos) y la violencia (pareja física, sexual e íntima) como variable de exposición. Realizamos análisis de regresión lineal para estimar la asociación entre la exposición y el resultado. En total, el 90 % de los AHSH y el 95 % de los mujeres transgénero y travestis experimentaron al menos una forma de violencia en los tres meses anteriores a la encuesta, y alrededor del 45 % de los mujeres transgénero y travestis experimentaron violencia sexual en el mismo período. Experimentar discriminación dentro de los ambientes de atención médica (instituciones o proveedores) se asoció negativamente con las prácticas de salud sexual. La discriminación y la violencia afectan negativamente las prácticas de salud sexual. La prevención contra el VIH y el cuidado a AHSH y mujeres transgénero y travestis deben escuchar sus experiencias y hacer frente a la discriminación y la violencia en esa población.

## Introduction

Adolescence is a challenging period of biopsychosocial development and transformation, a transitional phase from childhood to adult life ^1^ marked by conflicts, tensions, and ambiguities. Gender and sexually diverse adolescents and young people, such as gay men, men who have sex with men (AMSM) and *travestis* and transgender women (ATGW) face specific challenges as they live in prevailing heteronormative environments that significantly impact their physical and mental health ^2^. Studies have consistently shown that AMSM and ATGW engage in more risky behaviors and have worse health outcomes than cisgender heterosexual adolescents ^1^ ^,^ ^3^ ^,^ ^4^ ^,^ ^5^, especially regarding unsafe sexual practices (e.g., inconsistent use of condom), drug abuse, and traumatic sexual experiences ^6^ ^,^ ^7^. 

Studies have reported that adolescents and young adults represent a growing share of people living with HIV in Brazil and worldwide. Estimates suggest that around four million young people aged from 15 to 24 years currently live with HIV worldwide, 29% of which are aged 15-19 years ^8^. In Brazil, HIV cases among male adolescents aged 15-19 years increased by 496% from 2010 to 2019 ^9^. The response to HIV, including prevention, care, and treatment, remains a challenge among young Brazilians ^10^ ^,^ ^11^.

The prevalence of HIV is disproportionally higher among AMSM and ATGW than in the general population ^12^ ^,^ ^13^. Violence affects the lives of these people, making them more vulnerable to HIV infection. It can manifest itself in all forms of discrimination, social exclusion, and structural inequalities, such as poverty, racism, and gender inequality ^14^ ^,^ ^15^. Violence against AMSM and ATGW remains greatly underreported ^16^ despite being quite significant. Brazil has the highest homicide rate of TGW in the world ^17^, an expression of extreme violence resulting from intolerance against this population.

Adolescents in general (and AMSM and ATGW especially) are more vulnerable to HIV ^18^ ^,^ ^19^ due to several factors associated with adolescence. As adolescents engage self-discovery and experimentation, these may clash with poor knowledge on HIV/AIDS and misconceptions about HIV prevention. They encounter a gap between prevention rhetoric and practice and perceive themselves at low risk of infection, thus sharing social representations of HIV/AIDS that are distant to their reality (e.g., “an adult disease”, “disease of the 80s”). This population suffers from a lack of social systems to protect them against HIV as public policies fail to promote comprehensive healthcare and result in inadequate health services ^20^ that are ill-equipped to address adolescents and LGBTQIA+ adolescents’ specific needs and demands ^1^.

Violence is highly prevalent in adolescents’ lives ^21^ as they are either exposed to it directly as victims or indirectly as witnesses. Violence against adolescents is a major public health concern in Brazil ^22^ as homicide is the main cause of death in this age group ^16^. Although this remains an underreported phenomenon, adolescents are chronically exposed to violence and violent acts, which predominantly take place in their family environments and are mostly perpetrated by their family members and/or acquaintances. Violence is also highly prevalent in public environments - known as community violence ^23^. A study conducted with LGBTQIA+ people in Brazil using data from the Brazilian Information System for Notifiable Diseases (SINAN) between 2015 and 2017 ^24^ reported that around 55% of violence incidents occurred within the victims’ household, they were repetitive, and had been perpetrated by family members. Physical violence is its most common form, affecting mostly black and ATGW adolescents. Natarelli et al. ^1^ reported that physical violence against these populations usually involved violent disciplinary acts to punish the behavior of “unadjusted” teenagers and to discipline, manage, and control them. The then Brazilian President Jair Bolsonaro also encouraged this kind of punishment, stating: “...*if your son becomes gay, give him a beating and he will change his behavior*...” ^25^.

AMSM and ATGW suffer a syndemic between HIV, violence, and discrimination against them. These epidemics seem to reinforce each other in this population and should be understood on their interconnections and multiple interactions ^26^. Singer et al. ^26^ found a complex web of unfavorable social circumstances in HIV epidemics, violence, and drug use that, together, have adverse effects on living and health conditions of certain populations. Hence, we need to holistically address these phenomena.

In this study, we aimed to examine whether violence and discrimination experiences are associated with high-risk HIV behaviors and unsafe sexual practices among AMSM and ATGW. We also assessed differences by gender identity, sexual orientation, and race/skin color. We hypothesized that adolescents experiencing violence and discrimination (especially physical and sexual violence) engage more in risky sexual behaviors due to internalized stigmas toward their gender identity and/or sexual orientation.

## Methods

This is a cross-sectional study with baseline data from the PrEP1519 project. PrEP1519 is the first HIV pre-exposure prophylaxis (PrEP) demonstrative cohort study among key adolescent populations in Latin America, conducted in three large Brazilian capitals, Belo Horizonte (Minas Gerais State), Salvador (Bahia State), and São Paulo. The main objective of the study was to assess the effectiveness of daily oral PrEP to prevent HIV infection among high-risk AMSM and ATGW aged 15-19 years.

AMSM and ATGW aged 15-19 years who had at least one sexual intercourse with a cisgender man or TGW in the 12 months prior to this survey and had reported spending most of their time (i.e., living, studying or working) within the study site area were included. Use of alcohol or any other drug use at the time of the interview for enrollment was used as exclusion criterion.

The intervention consisted of (a) daily oral PrEP or (b) other non-PrEP HIV prevention methods. Participants were asked to choose either one. They answered a structured questionnaire so an interviewer could collect data at the participating clinic at which participants were enrolled in the study from 2018 to 2020. Further details on the complete methodology for the PrEP1519 project can be found in Dourado et al. ^27^.

Adolescents who had had no sexual intercourse in the three months prior to this study and/or who failed to answer this question were excluded from this study (n = 128; 12.6%).

### Outcome variable - sexual health practices

Participants’ sexual health practices were evaluated using a five-point frequency response Likert scale (1 - never; 2 - rarely; 3 - sometimes; 4 - often; and 5 - always) by asking the following questions: (a) During passive anal sex, did your partner(s) use a condom?; (b) During active anal sex, did you use a condom?; (c) I asked my partner to withdrawal; (d) I did not take the passive role in anal sex; (e) My partner and I were tested for HIV; (f) I had sex without penetration (*gouinage*); and (g) I did not have anal sex.

These questions were evaluated for both past steady and casual partnerships. A score of mean responses was used in our analysis to determine adolescents’ repertoire and their HIV-related sexual health practices. All questions that were related to sexual health practices referred to the prior three months before enrollment in this study.

### Exposure variables - violence and discrimination

Exposure to violence was assessed by three different sets of questions, including:

1) Lifetime intimate partner violence: (a) Did your partner threaten, frighten, or harass you?; (b) Did your partner give you drugs without your knowledge or consent?; (c) Did your partner slap, punch, kick, or push you?; (d) Did your partner force you to have sex or perform sexual acts unwillingly, in a degrading way, or using threats or coercion?; (e) Did your partner threaten/injure you with a firearm such as a handgun?; (f) Did your partner threaten/injure you with a knife or using any other object or weapon?; and (g) Did your partner damage or steal your personal possessions, work appliances, papers, valuable items, or any money or financial resources?

2) Physical violence: Did you suffer any type of physical violence in the last six months because of your sexual orientation or gender identity?

3) Sexual violence: Has anyone ever made you have sex by using force or the threat of force?

Exposure to discrimination based on sexual orientation or gender identity in the six preceding months was assessed by the following: (a) Not being hired for a job or being fired; (b) Being treated unfairly or not being allowed to enter shops or leisure spaces; (c) Being treated unfairly in a healthcare facility or by a health provider; (d) Being treated unfairly or marginalized by a teacher at a school/college/training program; (e) Being excluded or marginalized by peers at a school/college/training program; (f) Being excluded or marginalized from a group of friends; (g) Being excluded or marginalized by neighbors; (h) Being excluded or marginalized by their family; (i) Being excluded or marginalized in a religious space; (j) Being treated unfairly by police officers or staff; (k) Being treated unfairly at a public environment (e.g., shelters, city services, public transportation); (l) Being blackmailed or extorted for money; (m) Being harassed on social media or any other online space; and (n) Feeling unsafe and afraid in public spaces.

For all sets of questions, binary response variables were used (0 - never; 1 - at least once) and a cumulative score of violence per set of questions (1 - physical violence; 2 - sexual violence; 3 - Intimate partner violence; 4 - discrimination) and a global score for all these categories (0 to 24) were created.

### Covariables

Gender (cisgender man; TGW); sexual orientation (bisexual; heterosexual; and homosexual); race/skin color (white; black; and other); schooling (up to 11 years; 12 years or more); currently experiencing homelessness (yes; no); and engaging in transactional sex in the last three months (yes; no) were included as covariables.

In this research, we chose to group brown and black people as black, following the Brazilian Institute of Geography and Statistics (IBGE) and advocated by the Brazilian Black Movement.

### Statistical analyses

First, descriptive analyses were performed using measures of central tendency and dispersion for quantitative variables and proportions for categorical variables. These analyses were stratified by gender identity to assess the specific characteristics of ATGW.

A mean sexual practice score was used as the outcome and a calculated cumulative discrimination and violence experience score, as the exposure variable (by sets of questions and global score). Crude and adjusted analyses were performed using linear regression models to test the association between sexual practice and discrimination and violence scores. For the linear regression, sexual orientation (straight + bisexual; homosexual) and race/skin color (black; white + others) were recoded into binary variables. Homelessness was excluded as a variable in our adjusted model due to its low frequency in the studied sample (1.47%, n = 13). The transactional sex variable was also excluded due to its statistical insignificance. Thus, the analysis was adjusted for gender, race/skin color, and schooling even when p-value > 0.05 given their theoretical significance and importance in the literature. Lastly, the same analysis was conducted for the cumulative discrimination and violence score.

No collinearity between independent variables of the model was found. All analyses were performed in Stata 15 (https://www.stata.com).

### Ethical considerations

This study was approved by the Research Ethics Committee of the World Health Organization (Protocol ID: Fiotec-PrEP Adolescent Study) and research ethics committees at each higher education institution involved in the study (University of São Paulo - USP: #3,082,360; Federal University of Bahia - UFBA: #3,224,384; and Federal University of Minas Gerais - UFMG: #2,027,889).

An informed consent form was explained and signed by those adolescents aged 18 years or more. For those aged under 18 years, the study sites used different strategies. In Belo Horizonte, adolescents’ parents/legal guardians were asked to sign an informed consent form and adolescents, an assent form. In Salvador, adolescents’ parents/legal guardians were asked to sign an informed consent form and adolescents, an assent form or were asked to sign an assent form after a multidisciplinary team considered their situation as family estrangement or at risk of physical, psychological or moral violence due to their sexual and gender orientation. In São Paulo, adolescents were asked to sign an assent form.

All participants were informed that they could withdraw from this study at any time and that they were free to refuse answering any question they perceived as too personal, sensitive, or distressing.

## Results

Table 1 shows participants’ sociodemographic characteristics. Most participants (42.8%) lived in São Paulo, followed by Salvador (35.4%) and Belo Horizonte (21.7%). Participants’ mean age was 18.7 years and most identified with the male gender (92%). Around 70% of volunteers reported their skin color as black. They had a high level of education, 52% reported 12 or more years of schooling. Almost all claimed not being affected by homelessness (98.5%) and 16% reported transactional sex and exchanging sex for money, favors, and/or gifts in the preceding three months.

**Table 1:** Sociodemographic characteristics of participants in the PrEP1519 project, 2018-2020 (n = 884).

Sociodemographic characteristics	n (%)
Age	
Mean (SD)	18.75 (1.02)
Median (IQR)	18.95 (18.24-19.53)
Gender identity	
Cisgender men	812 (91.86)
Transgender Women/*Travestis*	72 (8.14)
Race/Skin color	
White	235 (26.58)
Black	618 (69.91)
Other (indigenous, yellows)	31 (3.51)
Sexual orientation	
Bisexual	223 (25.23)
Heterosexual	55 (6.22)
Homosexual	606 (68.55)
Homelessness	
Yes	13 (1.47)
No	871 (98.53)
Transactional sex (last 3 months)	
Yes	142 (16.10)
No	740 (83.90)
Schooling (years)	
Up to 11	419 (48.40)
12 or more	465 (52.60)
Study site	
Belo Horizonte	192 (21.72)
Salvador	313 (35.41)
São Paulo	379 (42.87)

When we assessed discrimination experience related to sexual orientation and gender identity, 23.7% of AMSM and 50% of ATGW reported experiencing discrimination six times or more; and a very small proportion (12.8% AMSM and 9.7% ATGW), never experienced discrimination (Table 2). Despite the small number of ATGW participants (n = 72), they experienced much more discrimination than AMSM, with a mean score of 5.45 versus 3.56, respectively.

**Table 2:** Experiences of discrimination and violence reported by participants in the PrEP1519 project, 2018-2020.

	AMSM	ATGW
	n (%)	n (%)
Discrimination experience score		
Mean (SD); min-max	3.56 (0.10); 0-15	5.45 (0.42); 0-14
Discrimination experience (occurrences)		
Never	104 (12.8)0	7 (9.70)
1-5	514 (63.50)	29 (40.30)
6-10	173 (21.30)	29 (40.30)
> 10	21 (2.40)	7 (9.70)
Total	812 (100.00)	72 (100.00)
Discrimination modality		
Being treated unfairly at the workplace	87 (12.46)	10 (16.95)
Not being hired for a job or being fired	110 (17.15)	27 (42.19)
Being treated unfairly or not allowed to enter stores/Leisure spaces	193 (25.03)	34 (47.89)
Being treated unfairly in a healthcare setting (facility or provider)	73 (9.44)	25 (36.23)
Being treated unfairly/Marginalized by a teacher at school/college/training program	134 (17.56)	21 (30.43)
Being excluded or marginalized by peers at school/College/Training program	323 (41.57)	35 (50.72)
Being excluded or marginalized from a group of friends	180 (23.08)	27 (39.13)
Being excluded or marginalized by neighbors	191 (24.33)	33 (48.53)
Being excluded or marginalized by their family	340 (43.48)	38 (55.07)
Being excluded or marginalized in a religious space	211 (27.91)	24 (36.36)
Being treated unfairly by police officers or staff	101 (13.67)	14 (21.54)
Being treated unfairly at a public environment (e.g., shelters, city services, public transportation)	122 (15.84)	23 (32.39)
Being blackmailed or extorted for money	44 (5.69)	5 (7.14)
Being harassed on social media or any other online space	235 (30.21)	29 (41.43)
Feeling unsafe and afraid in public spaces	554 (70.04)	48 (67.61)
Violence experience		
Physical assault in the last 6 months	35 (4.32)	15 (21.13)
Forced sex (ever)	203 (25.44)	33 (45.83)
Sex partner violence		
Threatened, frightened, or harassed	145 (17.86)	23 (31.94)
Given drugs without knowledge/Consent	19 (2.34)	7 (9.72)
Slapped, punched, kicked, or pushed	93 (11.45)	21 (29.17)
Forced/Degraded to have sex	86 (10.59)	15 (20.83)
Threatened/Injured with firearm	14 (1.72)	8 (11.11)
Threatened/Injured with any object (e.g., knife)	18 (2.22)	9 (12.50)
Damaged, stolen personal possessions	57 (7.02)	13 (18.06)
Global violence score		
Mean (SD); min-max	4.39 (0.13); 0-20	7.45 (0.55); 0-18
Global violence experience (occurrences)		
Never	84 (10.34)	4 (5.56)
1-5	474 (58.37)	24 (33.33)
6-10	188 (23.15)	28 (38.89)
> 10	66 (8.13)	16 (22.22)
Total	812 (100.00)	72 (100.00)

AMSM: adolescent men who have sex with men; ATGW: adolescent *travestis* and transgender women.

Note that the most reported setting for experiencing discrimination and marginalization included public spaces (70.04% AMSM and 67.61% ATGW), followed by family environments (43.48% and 55.07%). In school settings, participants reported slightly rarer discrimination by friends/colleagues (41.57% and 50.72%). We found a higher prevalence of discrimination among ATGW than AMSM for all settings in which they reported experiencing discrimination, except for public spaces.

Table 2 shows a high prevalence of violence, with 4.32% of AMSM and 21.13% of ATGW reporting physical assaults and 25.44% and 45.83% reporting forced sex experiences, respectively, which suggests violent sexual socialization within adolescents’ relationships. As for violence perpetrated by casual or steady partners, our evidence suggests that ATGW experience this type of violence at higher rates than other key adolescent populations.

We found a mean global violence score - the sum of physical, sexual, and partner violence and discrimination experiences - equal to 4.36 for AMSM and 7.45 for ATGW. Violence experience ratings exceed 10 in 8.13% of AMSM and 22.22% of ATGW (Table 2).

Table 3 shows that, in the three months prior to this survey, 49.25% of AMSM and 46.34% of ATGW had at least one steady partner and 68% and 70%, had casual partners, respectively.

The most reported sexual health practices with steady partners included condom use both in passive (25.5% reported always using condoms and 15% often) and active anal sex (30.2% always using condoms and 12.6% often). ATGW reported similar rates, 24.3% always used condoms and 13.5% did so often in passive anal sex and 27.2% (always) and 9.1% (often) in active anal sex. Sexual practices AMSM and ATGW never or rarely engage in with steady partners included abstaining from either anal (70.2% and 64.6%) or passive anal sex (63.6% and 86.5%).

**Table 3:** Sexual health practices with steady and casual partners in participants in the PrEP1519 project, 2018-2020.

	Steady partner in the last 3 months		Casual partners in the last 3 months	
	AMSM	ATGW	AMSM	ATGW
	n (%)	n (%)	n (%)	n (%)
Total	458 (49.25)	38 (46.34)	632 (68.47)	58 (70.70)
Sexual health practices score				
Mean (SD); min-max	2.80 (0.04); 1.0-5.0	2.46 (0.12); 1.0-3.6	2.73 (0.03); 1.0-5.0	2.43(0.10); 1.0-4.7
Condom use with passive anal sex				
Never	90 (26.00)	9 (24.30)	51 (10.40)	9 (16.60)
Rarely	31 (9.00)	5 (13.50)	29 (5.90)	4 (7.40)
Sometimes	84 (24.30)	9 (24.30)	102 (20.80)	7 (12.90)
Often	52 (15.00)	5 (13.50)	91(18.50)	12 (22.20)
Always	88 (25.50)	9 (24.30)	217 (44.30)	22 (40.70)
Condom use with active anal sex				
Never	82 (25.30)	3 (27.20)	55 (13.40)	3 (13.60)
Rarely	33 (10.20)	2 (18.20)	17 (4.10)	1 (4.50)
Sometimes	70 (21.60)	2 (18.20)	85 (20.70)	2 (9.00)
Often	41 (12.60)	1 (9.10)	76 (18.50)	2 (9.00)
Always	98 (30.20)	3 (27.20)	177 (43.10)	14 (63.60)
Withdrawal				
Never	167 (41.80)	20 (55.60)	204 (36.00)	21 (38.10)
Rarely	27 (6.70)	2 (5.50)	33 (5.80)	4 (7.30)
Sometimes	69 (17.30)	6 (16.60)	77 (13.50)	12 (21.80)
Often	32 (8.00)	1 (2.80)	57 (100)	6 (10.00)
Always	104 (26.00)	7 (19.40)	197 (34.60)	12 (21.80)
Avoid anal sex (passive)				
Never	224 (52.80)	31 (83.80)	287 (48.00)	40 (70.10)
Rarely	46 (10.80)	1 (2.70)	64 (10.70)	4 (7.00)
Sometimes	64 (15.00)	2 (5.40)	108 (18.10)	7 (12.30)
Often	27 (6.30)	1 (2.70)	47 (7.80)	3 (5.20)
Always	63 (14.80)	2 (5.40)	91 (15.20)	3 (5.20)
HIV testing of partners				
Never	226 (51.80)	18 (51.40)	434 (72.90)	39 (72.20)
Rarely	35 (8.00)	1 (2.80)	54 (9.00)	6 (11.10)
Sometimes	38 (8.70)	3 (8.50)	45 (7.50)	7 (12.90)
Often	51 (11.70)	1 (2.80)	24 (4.00)	1 (1.80)
Always	86 (19.70)	12 (34.30)	38 (6.40)	1 (1.80)
Non-penetrative sex				
Never	173 (38.10)	14 (38.90)	273 (43.60)	21 (38.10)
Rarely	69 (15.20)	7 (19.40)	85 (13.60)	5 (9.00)
Sometimes	124 (27.30)	9 (250)	151 (24.10)	18 (32.70)
Often	57 (12.50)	5 (13.90)	74 (11.80)	5 (9.00)
Always	31 (6.80)	1 (2.70)	43 (6.80)	6 (10.90)
Not having anal sex				
Never	234 (53.00)	21 (61.70)	316 (51.50)	26 (510)
Rarely	77 (17.20)	1 (2.90)	71 (11.60)	6 (11.70)
Sometimes	88 (19.90)	6 (17.60)	148 (24.10)	10 (19.60)
Often	20 (4.50)	2 (5.90)	41 (6.70)	6 (11.70)
Always	23 (5.20)	4 (11.70)	37 (6.00)	3 (5.90)

AMSM: adolescent men who have sex with men; ATGW: adolescent *travestis* and transgender women.

With casual partners, always or frequently engaged sexual practices included condom use in both passive and active anal sex among AMSM (62.8% and 61.6%) and ATGW (62.9% and 72.6%). Sexual practices in which AMSM and ATGW never or rarely engaged included HIV testing (81.9% and 83.3%) and abstaining from anal (63.1% and 62%) and passive anal sex (58.7% and 77.1%). We found mean sexual practice scores equal to 2.8 for AMSM and 2.46 for ATGW. Engagement with casual partners showed similar scores (2.73 and 2.43).

Table 4 shows the associations between different types of experienced discrimination and violence and sexual health practices. In the crude analysis, being threatened or injured with a firearm, not being hired for a job and being fired, being treated unfairly in a healthcare setting (facility or provider), being treated unfairly or marginalized at school/training program by a teacher or peers/friends, and being excluded or marginalized by a group of friends were negatively associated with sexual health practices (p < 0.05). In the adjusted model, only discrimination in healthcare services remained associated with the outcome of interest.

**Table 4:** Associations between experiences of discrimination and violence and sexual health practices among adolescent men who have sex with men (AMSM) and adolescente *travestis* and transgender women (ATGW) in the PrEP1519 project, 2018-2020.

	Crude analysis		Adjusted analysis *			
	b	p-value	b	p-value	95% CI	R²
Sexual partner violence						
Being threatened, frightened or harassed	-0.30	0.68	-0.13	0.85	-0.15; 0.12	0.01
Drug use without knowledge or consent	0.02	0.87	0.10	0.52	-0.22; 0.43	0.02
Physical violence	-0.10	0.20	-0.05	0.51	-0.22; 0.11	0.02
Forced/Degraded to have sex	0.42	0.63	0.07	0.40	-0.10; 0.25	0.02
Threatened/Injured with a firearm	-0.42	0.02	-0.32	0.07	-0.68; 0.33	0.02
Threatened/Injured with a knife or any other object or weapon	-0.26	0.11	-0.18	0.28	-0.51; 0.15	0.02
Damaged/Stolen personal possessions	-0.85	0.42	-0.03	0.74	-0.24; 0.17	0.01
Violence						
Forced sex	-0.03	0.61	-0.00	0.91	-0.13; 0.12	0.01
Physical assaults	-0.15	0.20	-0.65	0.60	-0.31; 0.18	0.01
Discrimination						
Not being hired or being fired	-0.17	0.03	-0.12	0.12	-0.28; 0.35	0.02
Being treated unfairly or not being allowed to enter shops or leisure spaces	-0.60	0.36	-0.03	0.62	-0.16; 0.09	0.02
Being treated unfairly in a healthcare facility or by a health provider	-0.24	0.00	-0.18	0.04	-0.36; -0.00	0.02
Being treated unfairly or marginalized by a teacher at school/College/Training program	-0.15	0.03	-0.12	0.09	-0.27; 0.02	0.02
Being excluded or marginalized by peers at school/College/Training program	-0.03	0.57	-0.01	0.84	-0.12; 0.10	0.02
Being excluded or marginalized from a group of friends	-0.13	0.04	-0.11	0.11	-0.24; 0.02	0.02
Being excluded or marginalized by neighbors	-0.61	0.35	-0.02	0.72	-0.15; 0.10	0.01
Being excluded or marginalized by their family	-0.09	0.10	-0.08	0.17	-0.19; 0.03	0.02
Being excluded or marginalized in a religious space	0.002	0.97	0.01	0.76	-0.11; 0.15	0,01
Being treated unfairly by police officers or police staff	-0.12	0.15	-0.96	0.26	-0.26; 0.07	0.01
Being treated unfairly at a public environment (e.g., shelters, city services, public transportation)	-0.15	0.06	-0.11	0.14	-0.27; 0.03	0.02
Being blackmailed or extorted for money	-0.07	0.53	-0.63	0.60	-0.30; 0.17	0.01
Feeling unsafe and afraid in public space	0.01	0.86	-0.00	0.98	-0.12; 0.12	0.01
Being harassed on social media or any other online space	-0.21	0.74	-0.00	0.88	-0.13; 0.11	0.01
Being treated unfairly/discriminated at a workplace	-0.17	0.06	-0.16	0.88	-0.34; 0.02	0.01

95%CI: 95% confidence interval.

* Adjusted for gender, sexual orientation, race/skin color, and schooling.

Table 5 shows the results of the crude and adjusted linear regression analyses according to violence subtypes and global scores. Regarding exposure to discrimination, our crude analysis showed negative regression coefficients, suggesting that these adolescents engaged more in unsafe sexual practices as they experienced more discrimination.

**Table 5:** Associations between violence subtypes and global score and sexual health practices among adolescent men who have sex with men (AMSM) and adolescente travestis and transgender women (ATGW) in the PrEP1519 project, 2018-2020.

	Coefficient	p-value	95%CI	R²
Crude analysis				
Gender (reference: male)	-0.33	0.002	-0.53; -0.12	0.01
Sexual orientation (reference: homosexual)	0.06	0.322	-0.06; 0.19	0.001
Race/skin color (reference: Black)	-0.09	0.134	-0.22; 0.03	0.003
Schooling (reference: < 12 years)	0.15	0.01	0.04; 0.26	0.01
Transactional sex (refence: no)	-0.06	0.428	-0.21; 0.89	0.000
Discrimination exposure	-0.02	0.03	-0.04; -0.002	0.005
Global (discrimination and violence) score	-0.01	0.03	-0.03; -0.0012	0.005
Sex partner violence	-0.03	0.27	-0.08; 0.02	0.001
Sexual violence	-0.03	0.61	-0.16; 0.096	0.0003
Physical violence (6 months)	-0.16	0.20	-0.40; 0.09	0.002
Adjusted analysis *				
Sex partner violence	-0.01	0.69	-0.06; 0.04	0.02
Sexual violence	-0.01	0.91	-0.13; 0.12	0.02
Physical violence (last 6 months)	-0.07	0.60	-0.31; 0.18	0.02
Discrimination exposure	-0.01	0.13	-0.03; 0.004	0.02
Global (discrimination and violence) score	-0.01	0.17	-0.03; 0.004	0.02

95%CI: 95% confidence intreval.

* Adjusted for gender, sexual orientation, race/skin color, and schooling.

When we analyzed the global violence score (the sum of discrimination and violence experience), we found participants engaged in the same risky behavior, suggesting the harmful effects of violence and discrimination on sexual health practices. However, our adjusted analysis found no association between discrimination and global (discrimination and violence) score and sexual health.

We found neither significant association between forms of violence and sexual health practices nor differences in exposure to violence and sexual health practices according to type of partner.

## Discussion

Almost all adolescents in our study experienced at least one recent instance of violence. Around 25% of AMSM and 50% of ATGW have experienced a form of sexual violence in their lifetime. Although we studied a small sample of ATGW participants, violence against this population cannot be neglected and should be made visible. More accurate public policy response require increased production of evidence, thus entailing further investigations.

We can interpret the high prevalence of sexual violence against ATGW as resulting from the dehumanization and sexual objectification of the trans body. We found the same pattern of discrimination and violence among adolescents in either steady or casual partnerships when we analyzed different settings. Violent and discriminatory interactions occur mainly in public spaces, followed by family environments and schools. These findings agree with the literature, showing the central role of family ^24^ and education environments ^28^ as the places in which violence and discrimination occur in these populations. Note that families and schools should configure environments in which adolescents feel welcomed and cared for and are free to socialize. Brazil has seen a setback in the protection of sexual and reproductive rights and its public policies have received strong support from an agenda of conventional customs, resulting in a weak support to comprehensive sexuality education programs in public schools ^29^.

As for sexual health practices, consistent with the combination prevention approach ^30^, people engage in a multiple array of health practices depending on situations and relationships. Certain care practices are more commonly used as a repertoire in both steady and casual partnerships such as condom use (the most widely used and promoted strategy), whereas other strategies remain scarcely used, such as HIV testing and other prevention approaches involving sexual risk management. Our findings point to repertoires that are mostly incorporated in these populations and indicate that other care strategies could be reinforced. Condom use is the predominant preventive method against sexually transmitted infections, and this population scarcely uses other possibly more effective strategies either in combination with condom use or without it. The array of care strategies offered to adults in health services should also be offered to young people so they can navigate and choose among different approaches and reduce risky sexual practices. We found an association between discrimination in healthcare settings and unsafe sexual practices. Thus, providing friendly and non-discriminatory healthcare services to all populations is essential.

We found that most associations between discrimination and violence exposure and sexual health practices were statistically insignificant after we adjusted our models. Only the association between health practices and discrimination in a healthcare setting (facility or provider) remained significant after adjustment. Thus, experiencing discrimination in health settings negatively affected sexual adolescents’ health practices. Discrimination and violence against LGBTQIA+ population can be a key social health determinant because it is associated with worse health indicators and care inequalities ^31^, thus becoming a barrier to healthcare access ^32^. Studies have pointed out that healthcare services commonly show LGBTphobia and that this population have to face conservative views, discriminatory attitudes, moral judgments, and negligence from healthcare providers, hindering their access to healthcare ^1^ ^,^ ^31^ ^,^ ^32^. Our data corroborates this finding and stresses that discriminatory experiences in healthcare facilities and from providers have adversely affected people and prevented them from incorporating sexual health practices. Although we are unable to ascertain a causal relationship between these phenomena, we must consider their impact within health services. Health providers, especially those working in HIV/AIDS care services, must be aware that violence in all its multiple forms is part of the everyday life of LGBTQIA+ populations and they should receive training to address it and report related incidents and injuries to the relevant authorities. We should also consider the challenge healthcare services face while addressing the needs and demands of adolescent population ^1^ ^,^ ^33^.

Note the high prevalence of discrimination and violence victimization in our adolescent populations. Very few reported not experiencing violence. This high prevalence of violence points to a need for more adaptive self-care strategies to fight violence, exclusion, discrimination, and trauma among key adolescent populations. Their psychosocial development differs from that of their cisgender heterosexual peers. Growing up as young gay men/MSM and TGW requires a great deal of resilience as they face more challenges to affirm their identity and place in the world than other people ^34^, which may potentially have an adverse psychological impact and result in worse health indicators.

Given this high prevalence of victimization, these adolescents’ vulnerability to violence alone is unable to explain their increased risk of HIV and STIs. Therefore, a multi-pronged approach may substantially contribute to the understanding of this phenomenon and guide potential actions to optimize care processes for this population. It is crucial to raise awareness of the discrimination and violence these adolescents face and the need to address it using an integrated approach to sexual health and other health outcomes, such as mental health and substance abuse.

## Limitations

Our study has some limitations. First, it involved privileged adolescents as they belong to a protection and care network, have accessed care services to receive PrEP, and received the benefits of care, when compared to other same-age peers who may never seek this type of service or remain unaware of available care. Our results may have underestimated the actual magnitude of the association between violence, discrimination, and sexual health practices in this population. Young people enrolled in the PrEP program live in areas with potentially more resources and show greater willingness to receive care, have increased awareness of their rights, and can navigate the healthcare system. Further investigations on this issue with other design approaches are necessary. Moreover, demand creation efforts need to focus on key adolescent populations in HIV/AIDS care services.

Our society must implement public policies to prevent and address violence against LGBTQIA+ people. Primary health care services (and especially HIV and other STI prevention services) must develop strategies to address the impact of violence and discrimination in healthcare. Good care in public services should be accessible to these adolescent populations who often lack solid and safe support to face exclusion due to LGBTphobia.

Notably, we ignored drug use, whether within or outside sexual relations. This aspect directly relates to violence and sexual healthcare and consists of a key component of the SAVA (Substance Abuse, Violence and AIDS) model. However, due to the complexity of the phenomenon of drug use (including its social contexts and patterns of use), we decided to ignore this aspect. Nevertheless, we recognize the increasing frequency and diversity of drugs used in sexual intercourse and the relevance of studying the relation between drug use and sexual health care. Consequently, we suggest further research on this topic to comprehensively and deeply understand this issue.

We should stress the low representation of participants aged under 18 years (around 10%) as a limitation of the study, impacting the potential generalizability of our results to younger AMSM and ATGW. We highlight the barriers to healthcare service access for those aged under 18 years linked to a lack of knowledge and distrust. Additionally, we must consider that one of our inclusion criteria (legal guardians’ authorization) may have hindered this population’s possibility of participation. Finally, note that, as a result of the PrEP1519 study and other factors, eligibility criteria for PrEP in Brazil has include adolescents aged from 15 years onward since 2022.
